# Debate: Young people are living in unprecedented times – too much chaos or too little resilience? Protecting young people in perilous times calls for bolstering multisystem resilience as well as mitigating risk

**DOI:** 10.1111/camh.70088

**Published:** 2026-03-23

**Authors:** Ann S. Masten

**Affiliations:** ^1^ University of Minnesota Twin Cities Minneapolis‐Saint Paul MN USA

**Keywords:** Multisystem resilience, disaster, war, pandemic, protective factors

## Abstract

Young people around the world are threatened by increasing chaos and danger from armed conflicts, natural disasters, climate change, forced displacement, disease epidemics, and related adversities. This perilous situation raises a critical question: Are we doing enough to protect and prepare young people to adapt and thrive? Developmental research on risk and resilience suggests that the answer to this question is no, but there is considerable evidence to guide practice and policy going forward. Both reducing risk and bolstering resilience are essential strategies for promoting positive outcomes in a generation of young people contending with rising global chaos. Moreover, evidence suggests that the resilience of children depends on the operations and interactions of many systems within and around them that are amenable to change. These include neurobiological and psychological adaptive systems and supportive relationships, as well as safe and effective schools and communities. Building resilience for the younger generation is an investment in the future resilience of societies.

During the first quarter of the 21st century, global threats to children and their support systems escalated in the wake of armed conflicts, forced displacement, natural disasters, famine, pollution, and infectious diseases, including the COVID‐19 pandemic (Masten, 2025; Sanson & Masten, 2024; Oberg & Hodges, 2026; Save the Children, 2025). The massive scale of these hazards begs the question of whether we are doing enough to protect and prepare the younger generation facing unprecedented chaos. Decades of developmental research suggest that the answer to this question is no, we are not doing enough to reduce risk or to build the resilience young people need to adapt and thrive. Nonetheless, resilience science offers considerable evidence to guide the way forward.

There have been other periods in global history of heightened threats that affect multiple systems crucial to the well‐being of children, including the period surrounding two world wars and the Spanish flu epidemic during the first half of the 20^th^ century. Then too, huge numbers of young people were killed, injured, displaced, orphaned, starved, and traumatized by violence, leading to an outpouring of humanitarian aid for children and the 1946 establishment of the United Nations International Children's Emergency Fund (UNICEF). Currently, the cascade of threats to human life and well‐being affecting millions of young people around the world is unfolding in a larger and more interconnected world population with extraordinary levels of high‐speed and far‐reaching global communication afforded by the internet, social media, and smart devices. There is growing awareness that many global threats, including climate‐related disasters, are not randomly distributed, but more likely to endanger the lives of vulnerable children already affected by poverty, malnutrition, maltreatment, marginalization, historical trauma, and other risks to healthy development (Masten, Tyrell, & Cicchetti, 2023).

Global response to the COVID‐19 pandemic illustrated the devastating effects of a cascading multisystem disaster as well as the capacity for human resilience through individual and collective actions (Masten, 2025; Masten & Motti‐Stefanidi, 2020). There were many adverse effects of the pandemic, including death and disabilities, as well as economic stress and disruptions in many of the most important systems that support the health, development, and well‐being of children. Yet, there was also evidence of resilience and recovery across multiple system levels. For example, immune systems were boosted by the rapid development and deployment of novel vaccines. In family systems, parents learned to juggle work at home with supervising children and new demands of distance work or learning. Personnel in schools and workplaces learned to do their jobs remotely, and tools like Zoom were utilized on a new scale. Hospitals figured out better ways to treat pandemic patients. Supply chains essential to public health were re‐invented and streamlined. Nonetheless, it was clear at the height of the pandemic that we were not adequately prepared for the challenges presented by this kind of multisystem catastrophe. Many people died or suffered as families, communities, and countries struggled to adapt to the effects of a global pandemic. News organizations and governments were able to document the roller‐coaster pattern of COVID effects on health in objective and compelling graphs illustrating fluctuations in new cases, hospitalizations, and deaths. There undoubtedly was a roller‐coaster pattern in the social and emotional functioning of individuals and populations during the pandemic as well, with fluctuating feelings of well‐being versus exhaustion or despair, but psychological patterns were virtually impossible to document (Masten, 2025). The pandemic was a global wake‐up call to improve our preparation and response capacity for complex multisystem disasters like pandemics, wars, and natural or technological disasters.

The knowledge accruing in developmental resilience science offers important clues for how to conceptualize and build resilience for young people. Resilience can be broadly defined as the capacity of a dynamic system to adapt successfully through multisystem processes to challenges that threaten the function, survival, or development of the system (Masten, 2025). Like other contemporary researchers who study resilience, I think it is important to adopt a definition of resilience that facilitates communication and collaboration across different disciplines and levels of analysis or intervention. Multisystem threats require multisystem solutions that integrate research, knowledge, and interventions across fields and systems.

Developmental resilience science spans over half a century, providing many clues about the key promotive and protective influences that foster competence, health, and well‐being as children grow and develop (Masten, 2025; Masten, Lucke, Nelson, & Stallworthy, 2021). Over time, accumulating evidence pointed to a set of ordinary, common, but powerful resilience factors associated with better outcomes among diverse young people threatened by different kinds of adverse experience. The factors on this ‘short list’ represent major drivers of adaptive capacity in young people for a variety of challenges in life that involve many processes and systems (Masten, 2025).

Initially, studies of resilience in childhood focused primarily on psychological and social factors associated with positive adjustment in diverse children. Prominent examples included effective caregiving or supportive relationships, a sense of belonging, good problem‐solving skills, self‐regulation skills, positive views of the self, optimism or faith, a sense of meaning or purpose, effective schools, family routines and traditions, and connections with safe and well‐functioning communities. More recently, as evidence accrued in other fields from the work of scholars focused on resilience from the perspective of families, schools, communities, or larger human social organizations, scholars noted striking parallels in characteristics associated with resilience (Masten et al., 2021). These findings suggested that key protective factors involved in resilience, such as supportive relationships or problem‐solving capabilities, might work synergistically across system levels – collectively as well as individually – to enhance human potential for survival and adaptation to adversities. Fundamental human adaptive systems that promote survival and successful adaptation to adversity may have co‐evolved (biologically and socioculturally) to work together. However, to date, there is more focus on the components of resilience than there is on multisystem resilience and how networks of adaptive systems may work together to enhance adaptive capacity.

There is a growing consensus among contemporary scholars that human resilience is multisystemic, multidimensional, and dynamic, changing with experience and development (Masten et al., 2023; Ungar & Theron, 2020). Children depend on many systems for both their current and future protection from harm because they are embedded in many interacting systems that influence their capacity to respond to challenges. Based on evidence from intervention and education studies, many aspects of resilience appear to be malleable. Families and educators play central roles in the current support and protection of children in their care, as well as nurturing the psychosocial and problem‐solving skills, motivation, and mindsets that children will need as they grow up in a given sociocultural environment. Children learn from engaging with challenges, gaining skills and self‐confidence from successful engagement, while also learning when and how to enlist help for overwhelming threats.

The resilience literature suggests three broad strategies for improving the odds for positive development in the context of risk and adversity (see Masten, 2025): (a) lowering risk (e.g., preventing premature birth, removing landmines, treating maternal depression, or monitoring exposure to disturbing media coverage); (b) boosting promotive resources (e.g., providing early childhood education, libraries, green spaces to play, or computers and internet connections for distance learning); and (c) enhancing, mobilizing, or restoring powerful protective factors (e.g., vaccinating to boost immunity, supporting attachment relationships, reuniting children with caregivers, or implementing mentoring programs). In the past, efforts to address threats posed by natural disasters, war, and other alarming threats to children were often focused more on trauma responses and how to address physical or mental health issues that arose after exposure than they were on proactively building competence and resilience at multiple‐system levels to support child adaptation, recovery, and development.

A number of the most powerful and malleable resilience factors for children, such as problem‐solving or self‐regulation skills, parenting quality, and school engagement, have broad effects on multiple aspects of behavioral, social, and academic competence and development for children at low risk as well as children experiencing adversity (Masten, 2025). These are strong candidates for public health policy and investment, serving to build the tools for better adjustment in most children while nurturing the collective capacity for resilience in the general population (Berkel et al., 2023; Dodge et al., 2024). For more specific threats and vulnerabilities at the individual, family, or community level, it is important that preparations and responses be tailored and sensitive to variations in the nature of past, present, and expected threats as well as developmental, contextual, and cultural differences. While there remains much to learn about the processes that foster current and future resilience in the lives of children, existing literature offers considerable guidance for multisystem thinking as well as practical approaches for protecting children from chaos while building their resilience capacity to adapt and thrive. Lowering risk and bolstering resilience in the younger generation also builds the resilience of their future societies.

## Funding information

No funders declared.

## Conflict of interest statement

The author declares no conflicts of interest.

## Ethics statement

No ethics approval was required for this debate article.

## Data Availability

Data sharing is not applicable to this article as no data sets were generated or analyzed for this debate article.
